# Multiparameter flow cytometric analysis of CD4 and CD8 T cell subsets in young and old people

**DOI:** 10.1186/1742-4933-5-6

**Published:** 2008-07-25

**Authors:** Sven Koch, Anis Larbi, Evelyna Derhovanessian, Dennis Özcelik, Elissaveta Naumova, Graham Pawelec

**Affiliations:** 1 Center for Medical Research (ZMF), University of Tübingen, Waldhörnlestrasse 22, 72072, Tübingen, Germany; 2 Central Laboratory of Clinical Immunology, University Hospital Alexandrovska, 1 Georgy Sofiisky Str., 1431, Sofia, Bulgaria; 3 Academic Medical Center, University of Amsterdam, Experimental Immunology, Meibergdreef 9, 1100 DD, Amsterdam, The Netherlands

## Abstract

**Background:**

T cell-mediated immunity in elderly people is compromised in ways reflected in the composition of the peripheral T cell pool. The advent of polychromatic flow cytometry has made analysis of cell subsets feasible in unprecedented detail.

**Results:**

Here we document shifts in subset distribution within naïve (N), central memory (CM) and effector memory (EM) cells defined by CD45RA and CCR7 expression in the elderly, additionally using the costimulatory receptors CD27 and CD28, as well as the coinhibitory receptors CD57 and KLRG-1, to further dissect these. Although differences between young and old were more marked in CD8 than in CD4 cells, a similar overall pattern prevailed in both. Thus, the use of all these markers together, and inclusion of assays of proliferation and cytokine secretion, may enable the construction of a differentiation scheme applicable to CD4 as well as CD8 cells, with the model (based on Romero et al.) suggesting the progression N→CM→EM1→EM2→pE1→pE2→EM4→EM3→E end-stage non-proliferative effector cells.

**Conclusion:**

Overall, the results suggest that both differences in subset distribution and differences between subsets are responsible for age-related changes in CD8 cells but that differences within rather than between subsets are more prominent for CD4 cells.

## Background

Numerous studies have established that many parameters of immunity are decreased in elderly people and suggest that these are likely to contribute to their increased susceptibility to infectious disease and poor responses to vaccination [1-3]. In particular, the ability to control disease caused by novel pathogens is greatly compromised; responses to previously-encountered pathogens are, however, also eventually eroded in the very elderly [4]. These findings could be explained in at least two mutually non-exclusive ways: 1) that each T cell from an elderly donor is in some way compromised in its function, or 2) the proportions of the different T cell subsets differ between young and old people, but the function of each cell type is the same regardless of donor age. There is evidence for both views in that single T cells from the elderly may, for example, show apparent defects in signal transduction and activation [5]. However, most earlier studies examined mixed cell populations and apparent differences could have been due to different proportions of cells in the test populations. Studies with monoclonal populations have indicated age-associated changes at the single cell level [6], but these were associated with culture not chronological age and their relevance to the in vivo situation remains open.

**Figure 1:**
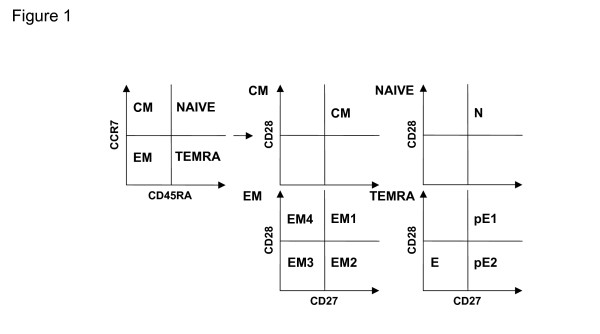
**Schematic model of the T cell differentiation subsets using the markers CCR7 and CD45RA and the co-stimulatory molecules CD27 and CD28**. With protein tyrosine phosphatase isoform CD45RA and the chemokine receptor CCR7, T cells can be subdivided into CD45RA+CCR7+ naïve (N), CD45RA-CCR7+ central memory (CM), CD45RA-CCR7- effector memory (EM) and CD45RA+CCR7- terminally differentiated effector memory (TEMRA) cells. The main subsets can be further subdivided by their expression of the co-stimulatory molecules TNF-family receptor CD27 and B7-family receptor CD28. Here, N and CM cells were defined as CD27+CD28+, whereas in EM and TEMRA further populations can be distinguished, which within EM cells are CD27+CD28+ (EM1), CD27+CD28- (EM2), CD27-CD28- (EM 3) and CD27-CD28+ (EM4), and within TEMRA are CD27+CD28+ (pE1,) CD27+CD28- (pE2), CD27-CD28- (E).

It is clear that age-associated thymic involution results in markedly decreased, but generally not absent, production of naïve T cells [7], while memory cell numbers increase in response to encountered pathogens [8]. The definition of "naïve" and "memory" cells in humans, is, however, controversial [9], and several models have been proposed based on cell surface expression of constellations of receptors and other molecules [10-12]. It must be borne in mind that these are all conceptual models and that lineages in this sense are not fixed; rather T cells are in a dynamic state of differentiation. One such model divides CD8 cells on the basis of their expression of the leukocyte common antigen isoform CD45RA and the chemokine receptor CCR7 into naïve (N; CD45RA+ CCR7+), "central" memory (CM; CD 45RA- CCR7+), "effector" memory (EM; CD45RA- CCR7-) and "terminally differentiated" effector memory (TEMRA; CD45RA+ CCR7-) cells [13]. Within each of these populations, the expression of the major T cell costimulatory receptors CD27, belonging to the TNF receptor family, and CD28, belonging to the B7 receptor family, has been applied to identify more (CD27- CD28-) or less (CD27+CD28+, CD27-CD28+ or CD27+CD28-) differentiated cells [14], schematically depicted in Figure 1. Differences between these T cell populations in young and old people have not yet been reported, but could provide useful data to discriminate between competing hypotheses to explain T cell changes in the elderly, namely whether these are caused entirely by altered frequencies of different cell subsets or by altered properties of cells within each of the subsets. This is a problem which has rendered interpretation of comparative data problematic over the years [5]. Here, we have employed polychromatic flow cytometry to investigate the frequencies of these different T cell subsets in young and old donors, examining both CD8 cells, as well as extending the above models to CD4 cells. Moreover, we have also included two other putative markers of highly differentiated T cells into this analysis, CD57 and Killer Lectin-like receptor G1 (KLRG1). The latter is an inhibitory C-type lectin-like receptor, a dimeric type-II trans-membrane glycoprotein with an extracellular domain homologous to C-type lectins and a cytoplasmic tail containing an immunoreceptor tyrosine-based inhibition motive (ITIM). KLRG1 is expressed on αβ T cells as well as NK cells [15] and γδ T cells [16] and was suggested to identify replicatively senescent cells [15,17]. CD57, the human natural killer-1 (HNK-1) glycoprotein, is found on many NK cells but also a subset of CD8 cells, where it has been reported to identify terminally differentiated T cells with reduced proliferative capacity [18,18]. In a more recent study, Ibegbu et al. showed that only CD57+ KLRG1+ double-positive T cells were impaired in their ability to proliferate [19]. The results presented here confirm that compared to the young, PBMC from the elderly contain decreased percentages naïve CD8 cells and increased TEMRA cells, while CD4 cells show similar but less marked trends. When dissected by their concurrent expression of CD27 and CD28, these subsets of T cells show further differences between young and old within subsets of CD8 cells and again fewer differences for CD4 cells. However, when CD57 and KLRG-1 are applied as additional markers of differentiation, greater age-associated disparities within CD4 subsets than CD8 subsets become apparent. These data are therefore consistent both with the existence of major differences between young and elderly donors residing in the altered frequencies of the different subsets and differences within those subsets, and re-emphasize that CD4 and CD8 cells show different age-associated changes in humans.

## Results

### Frequencies of N, CM, EM and TEMRA cells in young and elderly people

**Figure 2:**
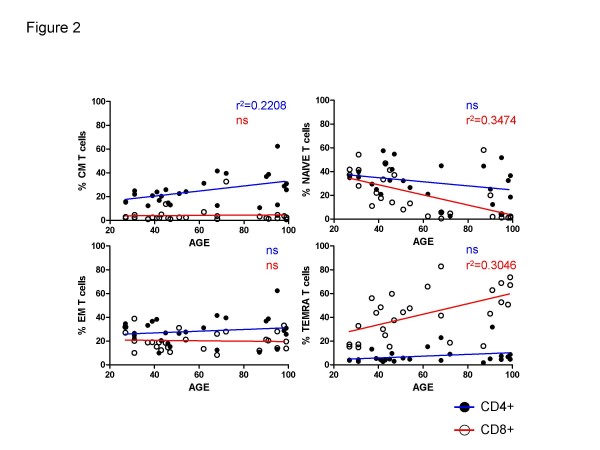
**Main T cells subsets identified in young and old by CD45RA and CCR7**. Frequencies of CD45RA+CCR7+ Naive, CD45RA-CCR7+ CM, CD45-CCR7- EM and CD45RA+CCR7- TEMRA CD8+ (open symbols) and CD4+ (filled symbols) at different ages in linear regression analysis.

PBMCs from the 37 donors tested were stained with antibodies to CD4, CD8, CD45RA and CCR7. The latter two markers were used to subdivide CD8+ or CD4+ T-cells into naïve and different memory subpopulations (Figure 1). Although as expected on the basis of the heterogeneous populations examined there is marked inter-individual variation even at the same age, an age-associated decrease in the frequency of naïve cells in both CD4+ and CD8+ cells was observed (Figure 2). However, this decrease achieved significance only for the CD8+ cells. There were no age-associated differences in the frequencies of either CD4+ or CD8+ EM cells, but, also as expected on the basis of previous reports for CD8+ cells, the proportion of CD8+ TEMRA cells increased significantly with age. Again, this was not seen for CD4+ cells. In contrast, there was a slight but significant increase in CD4+ CM cells which was not observed for CD8+ CM cells. This may suggest that whereas the loss of naïve CD8+ cells results in the accumulation of end-stage differentiated TEMRA cells in the elderly, for CD4+ cells, differentiation proceeds only to CM cells.

### Are the frequencies of subsets of CD8 cells defined by CD27 and CD28 expression different between young and old within the N, CM, EM and TEMRA populations?

**Figure 3:**
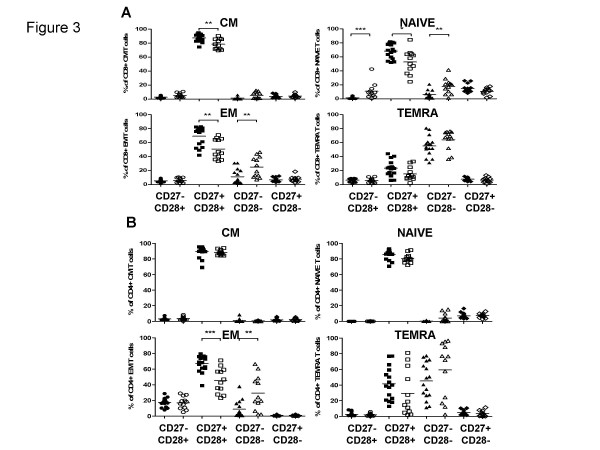
**Expression of co-stimulatory molecules CD27 and CD28**. The main T cell subsets were subdivided according to expression of the co-stimulatory molecules CD27 and CD28. Frequencies of CD27-CD28+, CD27+CD28+, CD27-CD28- and CD27+CD28- in N, CM, EM and TEMRA in (A) CD8+ and (B) CD4 T cells in young (filled symbols) and the elderly (open symbols) are shown. The naïve and CM cells were defined as CD27+CD28+, whereas the EM and TEMRA were divided into further subsets according to the model in Figure 1. Significant differences are indicated by asterisks, as in Figure 2.

An important question in establishing immune biomarkers of ageing is whether naïve cells are identical in young and old people and whether the age-associated differences are solely quantitative. The same question applies to the CM, EM and TEMRA subsets. Figure 1 shows the subdivisions of N, CM, EM and TEMRA cells according to the model of Romero et al. [14] defining 4 subsets of EM and 3 of TEMRA cells. According to this model, both N and CM cells are exclusively CD27 and CD28 double-positive. Here, we arbitrarily divided our donors into two groups, young (mean age = 40) and old (mean age = 87) and plot the data according to the Romero model. Figure 3A shows the results of CD27 and CD28 staining within the CD8+ N, CM, EM and TEMRA populations defined in Figure 1. Results with CD8 T cells from young people in general support the proposed model, i.e. naïve cells are almost mostly CD27+CD28+ although a minority does lack CD28, with very few CD27-CD28- or CD27+CD28-. There were no CD27-CD28+ CD8 cells. CM cells show similar phenotypes, whereas EM cells include increased fractions of CD27-CD28- double negative cells, consistent with some of them being more highly differentiated (having lost CD27 and CD28 expression). However, the majority remains CD27+CD28+. TEMRA cells, on the other hand, are mostly CD27-CD28-, with very few CD27+CD28- and CD27-CD28+ cells, but still retaining a sizeable population of CD27+CD28+ cells in most individuals. As previously noted for the proportions of N, CM, EM and TEMRA cells, there is a great deal of inter-individual variation in the distribution of these subsets.

Thus, the model of one subset of N, one of CM, and several of EM and TEMRA cells as defined by CD27 and CD28 expression does seem to hold up for CD8 cells from the young, as previously proposed [14]. However, the situation is not the same for CD8 cells from the elderly. The proportion of CD27+CD28+ cells within the naïve subset is significantly lower than in the young and the fraction of CD27-CD28+ cells significantly greater (Figure 3A). The proportion of CD27-CD28+ but not CD27+CD28- naïve cells is also significantly greater in the elderly than the young. For CM cells, differences between old and young reach significance only for the decrease in CD27+CD28+ cells. EM cells have decreased CD27+CD28+ and increased CD27-CD28- double negative cells in the elderly, with no significant differences between either the CD27-CD28+ or CD27+CD28- subset. Within the TEMRA cells, there are no significant differences between young and old in any subset, consistent with most TEMRA cells being as fully differentiated as they can be, regardless of the chronological age of the donor.

### Can subsets of CD4 cells also be distinguished on the basis of CD27 and CD28 expression and are they different between young and old within the N, CM, EM and TEMRA populations?

The model applied to CD8 cells above is not well established for the CD4 subsets. Age-associated changes in N, CM, EM and TEMRA subsets are also less well-documented for CD4 than CD8 cells. Data shown in Figure 2 had confirmed a decrease in naïve cells in the very elderly and increased proportions of CM cells. It remained possible that differences between young and old within each of these populations might be more marked. However, data shown in Figure 3B suggest that this is unlikely to be the case. There were no significant differences between N, CM or TEMRA cells in young and old, although here the EM cells of the elderly did show significant reductions in CD27+CD28+ cells and presumably reciprocal increases in CD27-CD28- cells.

### Can further subdivisions within subsets using CD57 and KLRG1 be informative in distinguishing between T cell subsets from young and old people?

#### Studies on CD8 cells

**Figure 4:**
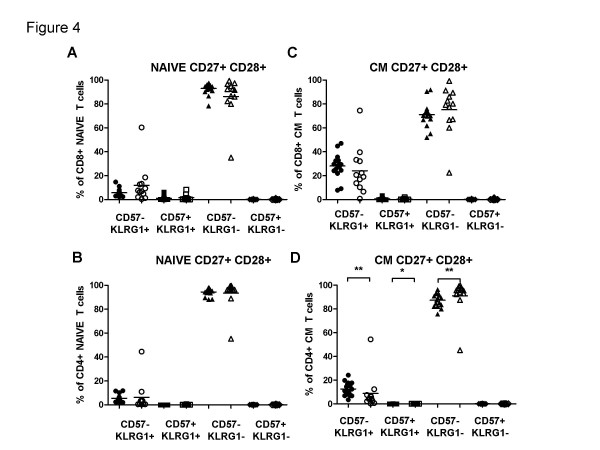
**Distribution of CD57 and KLRG1 in naive and CM T cells**. The CD27+CD28+ N and CD28+CD27+ CM (A and C) CD8+ and (B and D) CD4+ T cells in young (filled symbols) and the elderly (open symbols) were analysed for CD57 and KLRG1. Significant differences are indicated by asterisks, as in Figure 2.

Two cell surface NK receptors have been suggested to be markers of "senescent" T cells; CD57, which is expressed predominantly on CD8 cells, and KLRG1, which is of especial interest here because it is expressed on both CD4 as well as CD8 cells. A search for distinguishing features of the various subsets was first initiated in CD8 cells, as more likely to yield positive results. Figures. 4A, C examine expression of these markers on "fully" naïve (ie. CD27+CD28+) and CM cells of the same CD27+CD28+ phenotype, in the same groups of young and old donors. Almost all such naïve cells fail to express either CD57 or KLRG1 in either young or old donors, although there are signs of some cells starting to acquire KLRG1 (but not CD57) in the elderly. There is one outlying elderly individual in particular with a high level of KLRG1+ cells (and fewer KLRG1- cells) but even this donor's cells do not express CD57. Within the CD27+CD28+ CM cells, most individuals, whether young or old, now begin to express KLRG1, but still none express CD57, consistent with a differentiation pathway from naïve to CM marked by expression of KLRG1 but not CD57.

**Figure 5:**
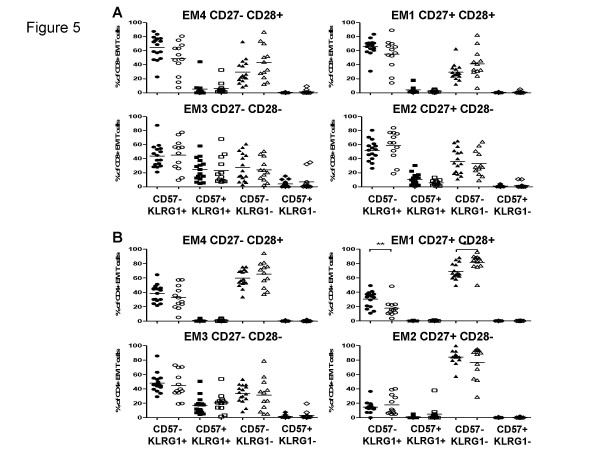
**Distribution of CD57 and KLRG1 in different EM T cell subsets**. According to the subset model depicted in Figure 1, the EM CD8+ (A) and CD4+ (B) were subdivided into CD27+CD28+ EM1, CD27+CD28- EM2, CD27-CD28- EM 3 and CD27-CD28+ EM4 T cells in young (filled symbols) and the elderly (open symbols) and analyzed for the expression CD57 and KLRG1. Significant differences are indicated by asterisks, as in Figure 2.

Data shown in Figure 5A address CD57 and KLRG1 expression by the 4 subsets of CD8+ EM cells. Essentially all cells within the EM1 subset remain CD57-, but the frequency of KLRG1+ cells is increased compared to the CM cells (compare Figure 4C). The EM2 subset, defined as CD27+CD28-, also includes very few CD57+ cells, although there is the hint of an increased occurrence of CD57+KLRG1+ cells. The EM4 subset (CD28-CD28+) appears very similar to the EM2 cells in this respect. However, the EM3 subset, defined as CD27-CD28-, contains a sizeable fraction of CD57+KLRG1+ cells. There are very few CD57+KLRG1- cells in any of the 4 subsets and those that are present are mostly in the EM3 subset. These findings are consistent with the proposal that the CD27-CD28- EM3 cells are the most highly differentiated of the 4 EM subsets. Figure 5 also documents a lack of any statistically significant differences between cells from the young or the elderly regarding any of these subsets. These data are consistent with differences between the young and old being due to altered frequencies of subsets and not differences within subsets and age.

**Figure 6:**
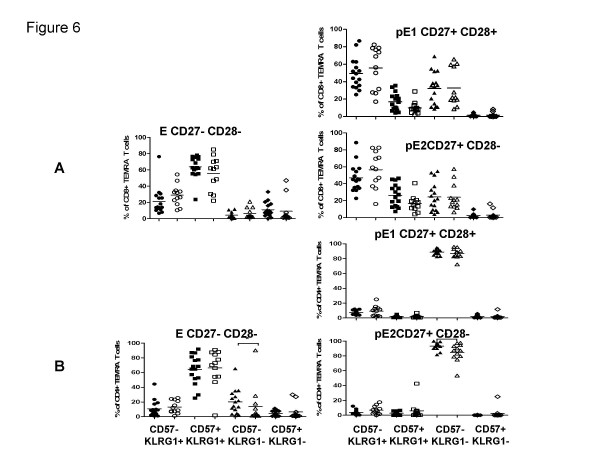
**Distribution of CD57 and KLRG1 in different TEMRA T cell subsets**. According to the subset model depicted in Figure 1, the EM CD8+ (A) and CD4+ (B) were subdivided into CD27+CD28+ pE1, CD27+CD28- pE2, CD27-CD28- E T cells in young (filled symbols) and the elderly (open symbols) and analyzed for the expression CD57 and KLRG1. Significant differences are indicated by asterisks, as in Figure 2.

Finally, the CD8 TEMRA cell subpopulations were examined in the same way (Figure 6A). Again, there are no differences between young and old. The number of KLRG1+CD57+ cells within the CD27-CD28-subset was greater than in the other two (designated pE1 and pE2 according to this model). It is notable that of all the subsets examined, CD57+KLRG1- cells appear only in what is proposed to be the most fully differentiated subset, the CD27-CD28- TEMRA subset.

#### Studies on CD4 cells

The final questions raised here were whether subdivisions of CD4 cells could be made in the same way as for CD8 cells, and whether these might reveal differences between the young and the elderly. Figures 4B and 4D show analogous data to Figures 4A and 4C but for CD4 rather than CD8 cells. The overall trend seems comparable: naïve cells contain very few but some KLRG1+CD57- cells but most cells are KLRG1-CD57-, whereas CM cells have slightly larger proportions of CD57-KLRG1+ but no CD57+ KLRG1- cells. Unlike CD8 cells, the CD4+ CM cells did show significant differences between young and old donors for all except the CD57+KLRG-1- cells. Data shown in Figure 5B in general support the same scheme for CD4 cells as applied to CD8 cells (compare Figure 5A for CD8). In this case there is a statistically significant difference between old and young EM1 cells, but unexpectedly with *fewer *CD57-KLRG1+ and more CD57-KLRG1- cells in the elderly than in the young. No other differences are statistically significant. Finally, Figure 6B shows data from CD4 TEMRA cells. Here, the differences between pE1/2 cells and the CD27-CD28- TEMRA cells are even more striking than seen for CD8 cells (compare Figure 6A). Thus, although neither pE1 nor pE2 cells contain CD57+KLRG1+ cells, the majority of cells in this most highly differentiated subset express both markers.

## Discussion

Differentiation stages of CD8 T cells can be ordered according to the expression of cell surface proteins, with a popular model describing naïve cells as CD45RA+ CCR7+ and memory cells as either CD45RA- CCR7+ (CM), CD45RA- CCR7- (EM) or CD45RA+ CCR7- (TEMRA). However, an absolute correlation between differentiation state and expression of these two surface molecules cannot be expected in such dynamic systems. Combinations of markers, however, may improve the ability to dissect out different subsets. Thus, naïve CD8 cells express CD27 and CD28, have long telomeres and extensive proliferative capacity [20]. The use of these markers may also not be sufficient, but the use of polychromatic flow cytometry for the first time allows constellations of markers to be used together for a finer definition of subsets at the single cell level. Here, we have explored the utility of combining the above markers with two others thought to be expressed by late-differentiated T cells, namely, CD57 and KLRG-1. All these markers together can be used to define subsets of memory cells with different properties, which we apply here not only for CD8 cells, but also for CD4 cells, which have not been extensively investigated previously [21]. Here, we have shown that subdivisions of EM and TEMRA cells on the basis of CD27 and CD28 expression can be further dissected by their expression of CD57 and KLRG-1, and moreover, that with the exception of CD57 expression, the same subsets can be defined in CD4 as well as CD8 cells. Finally, we have sought differences between young and old people within each of these newly-defined subsets.

We purposefully selected a heterogeneous cohort of young and old donors over a wide age range and from different European countries, so that any statistically significant differences found are likely to be robustly generally applicable, independently of genetic or environmental background. This is likely to be one reason for the large inter-individual variation observed in many of the tested parameters. Thus, it is all the more striking that the expected [22,23] age-associated reduction in the naïve cell population is clearly seen in CD8+ cells, as well as the increase in TEMRA cells (Figure 2). An age-associated reduction in naïve CD4+ cells was also observed, although this did not reach significance, and was accompanied by an increase in CM not TEMRA cells (Figure 2).

The expression of the costimulatory receptors CD27 and CD28 may be used to subdivide CD8 cells [14], demonstrating 4 populations of CD45RA- CCR7- EM cells and 3 of TEMRA (a CD27-CD28+ subset not being found in the latter). In general, in CD8 cells from young donors, our data confirm the presence of these subpopulations, although we found very few CD27-CD28+ or CD27+CD28- cells within either the EM or TEMRA subsets. Essentially the same pattern was found in cells from the elderly too, but interestingly within the EM but not within the TEMRA populations, there were significant differences between young and old for both CD27+CD28+ and CD27-CD28- cells (Figure 3A). This finding is consistent with the idea that subsets of TEMRA cells represent end-stage differentiated cells regardless of the age of the host, and which by definition cannot differentiate further.

Within the CD45RA+CCR7+ naïve CD8 cells, the elderly showed a reduction in CD27+CD28+ cells, an unexpected finding which could be explained by homeostatic proliferation of these cells with retention of naïve markers but loss of costimulatory receptors in the elderly. This would be likely to contribute to the poorer responses of naïve cells in old individuals, which has been demonstrated in TCR transgenic animal models, at least for CD4 cells [24]. Alternatively, these cells may not be "fully" mature but may in fact have re-acquired CCR7 as well as CD45RA, thus appearing naive. There is a precedent in the literature for such an event [25]. However, the picture with CD4 naïve cells was markedly different, with essentially all of them remaining CD27+CD28+, regardless of the age of the donor (Figure 3B). These data are consistent with the maintenance of a higher degree of both qualitative and quantitative integrity of the CD4 subset naïve potential than the CD8 with increasing donor age. However, as with CD8 cells, the CD4 subset also showed an increase of the CD27-CD28- EM3 cells at the expense of the CD27+CD28+ EM1 cells. This might also compromise the ability of previously activated effector memory cells to be reactivated, due to the lack of two major costimulatory receptors.

CD57 is a negative NK receptor also expressed on CD8 but not on CD4 cells, whereas KLRG-1 is a negative NK receptor expressed by both CD4 and CD8 cells [15]. Both have been dubbed "senescence" markers, and their absence from "fully" naïve and CM cells from the old as well as the young (Figure 4) is consistent with this. Both markers together identify the latest state of differentiation within the respective subsets. On CD8 cells, all 4 possible phenotypes are present (Figure 5A), whereas CD57 is not expressed on CD4 cells, not even from old donors, with two exceptions: EM3 cells are the only EM subset expressing any CD57, again independent of the age of the donor, but only by a minority of this subset and only by those cells already expressing KLRG-1 (Figure 5B). This suggests that a very small proportion of effector memory CD4 cells can indeed express CD57, and that EM3 cells are the most differentiated subset thereof, as with their CD8 counterparts. The other CD4 subset which expresses CD57 is the most differentiated of all: the CD27-CD28- TEMRA subset, again only on cells which are KLRG-1+ as well (Figure 6B).

By labelling whole PBMC with the stable membrane dye CFSE, cell division after stimulation can be tracked over 4–6 or more divisions. Applying this technique to CD4 and CD8 cells from young individuals assessed by the same surface markers as described above, and assuming that the more differentiated a cell is the less it can proliferate, we are in the process of accumulating data which are consistent with the model derived from the differential expression of CD45RA, CCR7, CD27, CD28, CD57 and KLRG-1 (data not shown). Reciprocally, at least for CD8 cells, the accumulation of perforin and Granzyme A is also consistent with the same differentiation scheme (data not shown). This scheme supports the notion that T cells differentiate sequentially through the stages N→CM→EM1→EM2→pE1→pE2→EM4→EM3→E and that the major but not the only differences, between cells from young and old donors reside in the relative proportions of these different subsets present. Nonetheless, using CD27 and CD28, age-associated differences primarily within CD8 but not CD4 subsets can be discerned, whereas using CD57 and KLRG-1 reveals such differences predominantly within the CD4 but not the CD8 subsets.

## Conclusion

Polychromatic flow cytometry is proving to be a powerful tool for examining changes in immune parameters in the elderly. The data provided here sought "lowest common denominator" changes in the distribution of both CD4 and CD8 subsets in different European populations over a range of ages from early middle aged to very old. By combining analyses using antibodies to CD27, CD28, CCR7, CD45RA, CD57 and KLRG-1, the results presented here confirm the robustness of the decrease in naïve and increase of late-differentiated CD8 cells with age, with a similar tendency in CD4 cells, in different populations with dissimilar genetic, nutritional and pathogen-exposure backgrounds. Combined with functional assays, this approach will facilitate the analysis of age-associated immune alterations in humans in unprecedented detail.

## Methods

### Donors

In order to encompass a broad (although exclusively Caucasian) population, cryopreserved PBMC were collected from several different European countries with a mean age of 40 (53% female) or 87 (66% female). Because this study was aimed at determining the most robust and reproducible differences between old and young donors, they were not rigorously selected for health status, nutrition, infection etc. They were from Italy, Sweden, Bulgaria and Germany, thus encompassing multiple genetic and environmental backgrounds. These donors were overtly healthy but were not SENIEUR-compliant, in order to study a more representative group of elderly. This approach obviously makes it more difficult to detect significant differences within such a heterogeneous group, but it is our belief that when significant differences do emerge in such a study, they are likely to be of more basic import than those seen only in highly selected subgroups. Thus, although infection with herpes viruses, especially CMV, is known to alter certain immune parameters, even this was also not taken into account in this study. Hence, we argue that any differences observed in this very heterogeneous group of subjects are more likely to reflect basic age-related alterations than influences of genetics, nutrition, infection etc.

### Antibodies

Direct immunofluorescence was performed with pre-titrated anti-CD4-PacificBlue, CD28-AlexaFluor700 (BioLegend, Biozol, Eching, Germany), CD8-APC-Cy7, CD27-APC CCR7-PE-Cy7 (Becton Dickinson, Heidelberg, Germany) and CD45RA-PE-Cy5.5 (Invitrogen, Karlsruhe, Germany). CD57 was from Immunotools, Friesoythe, Germany. For indirect immunofluorescence, anti-human KLRG1 (clone 13A2), kindly provided by Prof. H.P. Pircher, Freiburg, was used as primary antibody. As secondary antibody, Pacific Orange-goat anti-mouse IgG (Invitrogen) was used. For blocking, human immunoglobulin GAMUNEX (Bayer, Leverkusen, Germany) or mouse serum (Caltag/Invitrogen, Karlsruhe, Germany) were used. The cell viability was determined with Ethidium monoazide (EMA) (Invitrogen). All staining steps were performed in PFEA buffer (PBS, 2% FCS, 2 mM EDTA and 0.01% Azide).

### Staining

For each experiment, cells or mouse or hamster/rat κ-chain Comp Beads (Becton Dickinson) were stained with corresponding fluorochrome-labelled antibodies and incubated for 20 min at 4°C in the dark. As unstained negative controls we used negative Comp Beads (Becton Dickinson). After washing with PFEA, the cells or beads were resuspended in 200 μl PFEA and measured using an LSR-II flow cytometer and the acquisition software BD FACSDiva (Becton Dickinson). The spectral overlap between all channels was calculated automatically by the BD FACSDiva software, after measuring negative and single-colour controls. For additional data analysis, Flowjo 7.2.2 (Treestar Inc, San Carlos, CA) was used. Careful control experiments indicated that cryopreservation and thawing of the PBMC did not alter the pattern of staining observed for the major subsets studied here (Larbi et al., manuscript in preparation).

**Figure 7:**
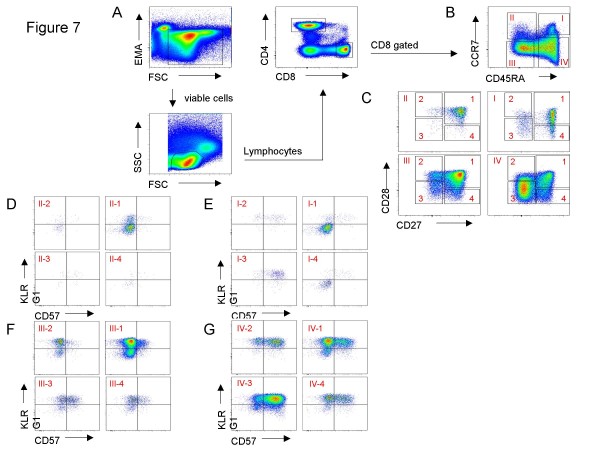
**Gating strategy for CD8+ T cells**. (A) Viable Lymphocytes were gated and then selected for either CD8+ or CD4+ T cells. (B) The CD8+ T cells were subdivided into the main T cell subsets using CD45RA and CCR7. (C) The CD45RA+CCR7+ N, CD45RA-CCR7+ CM, CD45-CCR7- EM and CD45RA+ CCR7- TEMRA CD8+ T cells were plotted against CD27 and CD28. According to the subset model (Figure 1) the different CD27 and CD28 dependent subpopulations (D) CM, (E) N, (F) EM and (G) TEMRA subsets were analyzed for CD57 and KLRG1.

**Figure 8:**
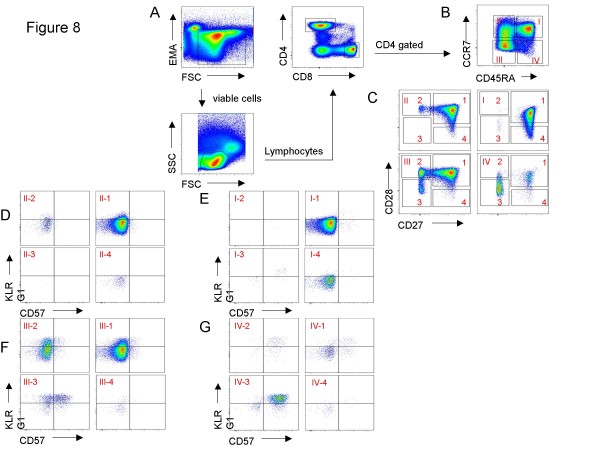
**Gating strategy for CD4+ T cells**. (A) Viable Lymphocytes were gated and then selected for either CD8+ or CD4+ T cells. (B) The CD4+ T cells were subdivided into the main T cell subsets using CD45RA and CCR7. (C) The CD45RA+CCR7+ N, CD45RA-CCR7+ CM, CD45-CCR7- EM and CD45RA+ CCR7- TEMRA CD4+ T cells were plotted against CD27 and CD28. According to the subset model (Figure 1) the different CD27 and CD28 dependent subpopulations (D) CM, (E) N, (F) EM and (G) TEMRA subsets were analyzed for CD57 and KLRG1.

For data analysis the following populations were gated in sequence. The first gate was Time vs. SSC-A to detect differences in the flow. To exclude dead cells, the EMA-negative population was selected in FSC-A vs. EMA to identify lymphocytes within the FSC-A vs. SSC-A dot plot for further analysis. Different fluorochrome-stained populations were highlighted by XY-quadrants and gates to obtain the corresponding statistics including counted events, percentage of parent, percentage of total, mean and median fluorescence intensity. Gating strategy is shown in Figures 7 and 8.

### Statistics

All statistical analysis were performed with Graphpad Prism 4.03 and 5.0. Non-parametric Mann-Whitney *U *test was used for comparison of two independent groups.

## Competing interests

The authors declare that they have no competing interests.

## Authors' contributions

SK helped design the study, carried out the flow cytometry studies, performed the statistical analysis and helped draft the manuscript, in contribution to work included in his PhD thesis. AL & ED participated in the design of the study and analysis of the flow cytometry data, and helped draft the manuscript. DO performed some of the flow cytometry. EN provided samples and data on donors. GP conceived the study, participated in its design and coordination, and drafted the manuscript. All authors read and approved the final manuscript.
